# Bioactivities of *Garcinia kola* enzymatic hydrolysates at different enzyme–substrate ratios

**DOI:** 10.1186/s13568-023-01583-2

**Published:** 2023-07-26

**Authors:** Salmat Adenike Salami, Olukemi Adetutu Osukoya, Olusola Bolaji Adewale, Oludele Odekanyin, Tajudeen Olabisi Obafemi, Adenike Kuku

**Affiliations:** 1 Biochemistry Programme, Department of Chemical Sciences, Afe Babalola University, Ado-Ekiti, Nigeria; 2 Department of Biochemistry and Molecular Biology, Obafemi Awolowo University, Ile-Ife, Nigeria

**Keywords:** Bioactive peptide, *Garcinia kola*, Protein hydrolysate, Plant dietary protein, Processing Oxidative stress, Inflammation

## Abstract

Natural products, such as enzymatic hydrolysates and bioactive peptides from dietary sources, are safe alternatives to synthetic compounds linked to various deleterious effects. The purpose of this study is to determine the in vitro bioactivities (antioxidant and anti-inflammatory activities) of *Garcinia kola* seeds enzymatic hydrolysates (GKPHs) at different enzyme (pepsin)-substrate ratios. *G. kola* protein, isolated by alkaline solubilization and acid precipitation, was hydrolyzed with pepsin at varying enzyme–substrate (E:S) ratios. The antioxidant parameters investigated include 1,1-diphenyl-2-picrylhydrazyl (DPPH)-radical scavenging, hydrogen peroxide scavenging and ferrous ion (Fe^2+^) chelating activities. For anti-inflammatory properties, membrane stabilization and protein denaturation activities tests were used. GKPH produced at 1:32 had the highest degree of hydrolysis (66.27 ± 4.21%). All GKPHs had excellent in vitro anti-inflammatory properties. However, only enzymatic hydrolysates produced at 1:16 (E:S) ratio chelated iron (II) and as well had the highest percentage hemolysis inhibition of 84.45 ± 0.007%, percentage protein denaturation inhibition of 53.36 ± 0.01% at maximum concentration and exhibited highest DPPH scavenging activity (87.24 ± 0.10%). The enzymatic hydrolysates had excellent solubility, emulsifying and foaming properties. It could be deduced from this study that pepsin at a ratio of 1:16 of *G. kola* protein produced the most effective enzymatic hydrolysates in terms of their antioxidant and anti-inflammatory activities. *G. kola* pepsin enzymatic hydrolysates, thus, have potential in development as functional foods and as therapeutics pharmaceutical industries in the management of diseases associated with oxidative stress and inflammation owing to their excellent functional, antioxidant and anti-inflammatory properties.

## Introduction

Oxidative stress reflects a disparity between oxidants generation and antioxidant capacity, causing reactive oxygen species (ROS) to amass in the system. ROS has been associated with various diseases, including inflammation, ageing, diabetes, neurodegenerative, cardiovascular and cancer diseases (Li et al. 2016). The excess ROS, produced either by exogenous or endogenous sources, can oxidize biological molecules, modify proteins and genes, leading to lipid, protein and DNA damage (Akhigbe and Ajayi 2021), which is the onset of many chronic diseases. Also, oxidative stress can activate several transcription factors, causing the upregulation of some genes involved in inflammatory pathways, thereby leading to various chronic diseases (Hussain et al. 2016).

Many chemical antioxidants, such as butylated hydroxytoluene (BHT), butylated hydroxy anisole (BHA), tertbutyl hydro-quinone (TBHQ), and propyl gallate (PG), have been used in managing diseases connected to oxidative stress. However, these chemicals have adverse side effects such as allergic reactions on the skin, carcinogenesis, hormone function interference, liver, thyroid and kidney problems and blood coagulation (Baur et al. 2001). Also, synthetic anti-inflammatory drugs belonging to steroidal and non-steroidal anti-inflammatory drugs (NSAIDs) have been linked to several side effects such as gastrointestinal disorders, cardiovascular disorders, osteoporosis, renal failure and diabetes (Oladokun et al. 2019). Hence, it is necessary to explore natural products, such as bioactive protein/enzymatic hydrolysates from dietary sources, as safe alternatives to synthetic products in managing diseases (Sang et al. 2019).

Bioactive protein hydrolysates are heterogeneous combinations of peptides, oligopeptides and free amino acids produced by partial or extensive hydrolysis of proteins that positively impact body functions or conditions and may influence health (Sánchez and Vázquez 2017). The functions of protein hydrolysates are interconnected with the biopeptides (3–50 amino acid residues in length) present in them, while their activities depend on the collective residues present, class of N- and C-terminal residue, peptide chain length, amount of charge on the amino acids forming the peptide and the hydrophobic/hydrophilic characteristics of the primary structure (Chauhan and Kanwar 2020). Protein hydrolysates possess potent bioactivities, notably antioxidant, antimicrobial, immunomodulatory, anti-inflammatory, anti-hypertensive, anti-human immunodeficiency virus (anti-HIV) and opioid activities (Chauhan and Kanwar 2020). For instance, protein hydrolysates with antioxidant activity can inhibit and scavenge free radicals, alleviating oxidative stress.

Naturally occurring antioxidant peptides and those derived from protein hydrolysis are considered novel and potential dietary ingredients that promote human health (Osukoya et al. 2020). Protein hydrolysates from African breadfruit (Osukoya et al. 2020), calabash nutmeg seeds (Osukoya et al. 2021), Mormyrids muscle (Jerome and Olayemi 2019), grass turtle (Islam et al. 2021), rice (Zhang et al. 2020) and sweet potato (Zhang et al. 2012) amongst others had shown potent antioxidant properties. Some examples of plant protein hydrolysates with anti-inflammatory properties include those obtained from yellow field pea seeds (Ndiaye et al. 2012), chia seeds (Chan-Zapata et al. 2019), hemp seeds (Rodriguez-Martin et al. 2020) and white carob seed cotyledon (Cattaneo et al. 2014).

*Garcinia kola* Heckel belongs to the family *Clusiaceae*, and it is commonly called bitter kola or bitter cola. In Nigeria, it is locally referred to as *namijin goro*, *orogbo* and *adu* by the Hausa, Yoruba and Ibo tribes, respectively. In folklore medicine, extracts of these seeds are used as a remedy for laryngitis, cough, asthma (Iwu and Igboko 1982), liver disorders (Farombi et al. 2005), hepatitis, bronchitis and gonorrhoea (Okojie et al. 2009). The seed is also used as an antidote to food-borne disorders and snakebites. It has been found efficacious in increasing low sperm count and as an aphrodisiac (Daramola and Adegoke 2011). The seed coat is also used as a hop substitute in several indigenous alcoholic drinks and a flavour enhancer in the beverage industry (Buba et al. 2016). Extracts of *G. kola* seed have been shown to have antioxidant and anti-inflammatory effects in type 1 diabetes mellitus rats (Idris et al. 2020).

Protein hydrolysates, derived from plants and animals particularly, with physiological activities and good functional properties are employed in food and pharmaceutical industries (Osukoya et al. 2021). Therefore, awareness of the functional properties and bioactivities of *G. kola* seed protein/enzymatic hydrolysates could increase their economic importance and market value as food or therapeutic ingredients in pharmaceutical industries in managing oxidative stress and inflammation-related disorders. Earlier, in our laboratory, protein hydrolysates were prepared from *G. kola* seeds using the proteolytic enzymes: trypsin, pancreatin and pepsin for the hydrolysis. Investigation revealed that the protein hydrolysates produced by pepsin hydrolysis possessed the highest bioactivity (Osukoya et al. 2022). Therefore, this study aimed to determine the effect of varying enzyme (pepsin): substrate ratio on the bioactivities (antioxidant and anti-inflammatory activities) of *G. kola* seeds enzymatic hydrolysates.

## Material and methods

### Materials

Fresh matured *G. kola* seeds were purchased from a local farm in Ijurin-Ekiti, Ekiti State, Nigeria. It was identified at the IFE herbarium, Department of Botany, Obafemi Awolowo University, Ile-Ife (voucher number: IFE-17998), where a copy was deposited. Pepsin (> 250 U/g), Folin–Ciocalteu’s phenol reagent, DPPH and trichloroacetic acid were purchased from Sigma-Aldrich, USA. All other reagents were of analytical grade and purchased from Loba Chemie, Mumbai.

### Preparation of *G. kola* seed protein isolate

*Garcinia kola* seeds were dehusked, chopped into small pieces and ground into a fine powder with an electric blender. The powdered seed was homogenized in 0.1 M NaOH (1/5 w/v ratio), stirred at room temperature for 4 h and kept at 4 °C overnight as described by Osukoya et al. (2021). The homogenate was clarified by centrifugation at 4000 rpm for 15 min, and the pellet was discarded while the supernatant containing soluble protein was precipitated with 2 M HCl to pH 4.0 (acid precipitation), left at 4 °C overnight and centrifuged. The pellet (protein isolate) obtained was freeze-dried and stored in an air-tight container at − 20 °C until further use. Protein concentration was determined according to Lowry et al. (1951) method using 1 mg/ml bovine serum albumin (BSA) as protein standard.

### Proximate analysis of *G. kola* seed and protein isolate

The moisture, crude protein, crude fibre, ash and crude fat present in fresh seeds of *G. kola* seed and protein isolate were determined as described in AOAC (2005). Carbohydrate content was estimated by the difference in nitrogen-free extract (NFE).

### Enzymatic hydrolysis of *G. kola* seed protein isolate

*Garcinia kola* protein isolate (GKPI) was hydrolyzed using pepsin at its optimal condition, 0.1 M glycine–HCl buffer (pH 2.0) as described by Osukoya et al. (2021). GKPI (4 mg/ml) in 0.1 M glycine–HCl buffer, pH 2.0, was digested with 1 mg/ml pepsin in the appropriate buffer using an enzyme: substrate ratio of 1:8, 1:16 and 1:32 (v/v). The mixtures, incubated at 37 °C with constant stirring and pH maintained at pH 2.0 using either glycine buffer or 6 M HCl for 4 h, were transferred to a boiling water bath at 100 °C for 10 min to inactivate the enzyme and immediately kept on ice. Protein concentration was determined using Lowry method.

### Physicochemical properties of *G. kola* seed enzymatic hydrolysates (GKPHs)

#### Degree of hydrolysis of GKPHs

A 20% (w/v) trichloroacetic acid (TCA) was added to an equal volume of GKPH and kept for 30 min at 4 °C. This mixture was centrifuged (3000 rpm, 10 min) to obtain 10% TCA-soluble proteins. The supernatants were assessed for protein content using Lowry’s method. Degree of hydrolysis (DH) was estimated as follows:

$$\% \;{\text{DH}} = \frac{{10\% \;{\text{TCA}} - {\text{soluble}}\;{\text{protein}}}}{{{\text{Total}}\;{\text{protein}}\;{\text{content}}\;{\text{in}}\;{\text{sample}}}} \times 100.$$

#### Peptide chain length estimation of GKPHs

The average peptide chain lengths (PCL) of GKPHs were estimated from the various values obtained from the DH and calculated as follows:

$${\text{PCL}} = 100/\% {\text{DH}}{.}$$

#### Amino acid composition of *G. kola* seed protein isolate and enzymatic hydrolysates

Amino acid analysis was determined as described by AOAC (2006) with modifications. The samples (GKPI and GKPH) were processed by drying, defatting, hydrolyzing and evaporating before loading onto the Applied Biosystems PTH Amino Acid Analyzer.

### In vitro antioxidant assays of *G. kola* seed enzymatic hydrolysates (GKPHs)

#### Determination of 1,1-diphenyl-2-picrylhydrazyl (DPPH) scavenging activity of GKPHs

The hydrogen or radical scavenging properties of GKPHs was determined by the stable radical DPPH method described by Zhu et al. (2008) with slight modifications. An equal volume of freshly prepared DPPH solution containing 0.25 mM DPPH in 95% methanol was mixed with an equal volume of varying concentrations (0.2, 0.4, 0.6, 0.8, and 1.0 mg/ml) of the standard and test samples in a 96-well plate. The mixture was incubated for 30 min in the dark at room temperature, and the absorbance was taken at 517 nm using a microplate reader. (Methanol replaced sample in blank, and ascorbic acid used as standard). The percentage inhibition of the standard and sample was calculated using this formula:

$$\% \;{\text{DPPH}}\;{\text{inhibition}} = \frac{{A_{blank} {-} A_{sample} }}{{A_{blank} }} \times 100.$$

#### Ferrous ion chelating ability assay of GKPHs

The in vitro Fe^2+^-chelating ability of GKPHs was determined as described by Osukoya et al. (2021). Aqueous FeSO_4_ (900 µl, 500 µM) and 150 µl of each protein hydrolysate were mixed and incubated for 5 min at room temperature. This was followed by the addition of 78 µl of 1,10-phenanthroline (0.2% w/v, ethanol). The absorbance of the orange-colored solution was immediately read at 510 nm. Glutathione and ascorbic acid were used as standard, while the blank contained distilled water instead of the sample. The in vitro Fe^2+^-chelating ability of the protein hydrolysate was calculated using the formula:

$$\% \;{\text{Chelating}}\;{\text{ability}} = \frac{{A_{blank} {-} A_{sample} }}{{A_{blank} }} \times 100.$$

#### Hydrogen peroxide (H_2_O_2_) scavenging ability of GKPHs

H_2_O_2_ scavenging ability of GKPHs was determined according to the method described by Fan et al. (2012) with slight modifications. A solution of 20 mM H_2_O_2_ was prepared in PBS, pH 7.2. Varying concentrations of GKPHs (1000 µl) were mixed with 2000 µl of 20 mM H_2_O_2_ and incubated for 10 min. Absorbance was taken at 230 nm using a UV–VIS spectrophotometer. Glutathione and ascorbic acid were used as standard, while the blank solution contained PBS and H_2_O_2_. The percentage inhibition of the standard and sample was calculated using the formula:

$$\% \;{\text{Inhibition}} = \frac{{A_{blank} {-} A_{sample} }}{{A_{blank} }} \times 100.$$

### In vitro anti-inflammatory assays of *G. kola* seed enzymatic hydrolysates (GKPHs)

#### Membrane stabilization method

Preparation of human red blood cells suspension: About 5 ml of blood (supplied by one of the authors) was mixed with an equal volume of Alsever solution (2.0% d-glucose, 0.8% sodium citrate, 0.05% citric acid and 0.42% sodium chloride). The blood was centrifuged at 3000 rpm for 15 min. The supernatant was discarded while the pellet containing the packed red blood cell was collected. The red blood cell was washed three times with isosaline solution (0.9% NaCl), and 10% (v/v) cell suspension was prepared.

Human red blood cell membrane stabilization test of *G. kola* seed enzymatic hydrolysates: Human red blood cell membrane stabilization by GKPHs was determined according to the method described by Javed et al. (2020). Various concentrations of hydrolysates were prepared in distilled water to a final volume of 1 ml, and to each concentration, 1 ml of phosphate buffer (0.1 M, pH 8.0), 2 ml of hyposaline (0.36% NaCl) and 0.5 ml of the prepared human red blood cell suspension were added. The mixture was incubated for 30 min at 37 °C and centrifuged at 3000 rpm for 20 min. Distilled water and 2 mg/ml acetylsalicylic acid were used as control and standard, respectively. The haemoglobin content in the supernatant solution was estimated spectrophotometrically at 560 nm. Hence, % hemolysis inhibition was calculated thus:

$$\% \;{\text{Hemolysis}}\;{\text{Inhibition}} = \frac{{A_{blank} {-} A_{sample} }}{{A_{blank} }} \times 100.$$

#### Protein denaturation method

Egg albumin was collected from a fresh hen’s egg. Inhibition of protein denaturation by GKPHs was evaluated as described by Ullah et al. (2014). Different concentrations of GKPHs were prepared using distilled water to a final volume of 2 ml. A 2.8 ml of phosphate buffer saline (0.1 M PBS, pH 6.4) and 0.2 ml of egg albumin was added such that the volume of the reaction mixture was 5 ml, incubated for 15 min at 37 °C, and heated at 70 °C for 5 min. Distilled water and 2 mg/ml acetylsalicylic acid were used as control and standard, respectively. After cooling, the absorbance was measured at 660 nm using PBS as blank. The percentage inhibition of protein denaturation was calculated thus:

$$\% \;{\text{Denaturation}}\;{\text{Inhibition}} = \frac{{A_{blank} {-} A_{sample} }}{{A_{blank} }} \times 100.$$

### Functional properties of *G. kola* seed enzymatic hydrolysate (GKPH)

The GKPH with the highest in vitro antioxidant activity was investigated for its functional properties according to the method of Osukoya et al. (2021).

#### Protein solubility of GKPH

GKPH (5 ml of 1 mg/ml) was dissolved in distilled water (20 ml) and stirred at room temperature for 25 min. Their pH was adjusted to pH from 2 to 12 with 0.5 M HCl or 0.5 M NaOH followed by centrifugation at 3000 rpm for 10 min. The protein content of the supernatant was determined using Lowry method, and % solubility was calculated as:

$$\% \; {\text{Solubility}} = \frac{{{\text{Protein}}\;{\text{content}}\;{\text{in}}\;{\text{supernatant}}}}{{{\text{Total}}\;{\text{protein}}\;{\text{content}}\;{\text{in}}\;{\text{sample}}}} \times 100.$$

#### Foaming properties of GKPH

The foaming properties of GKPH were investigated by dissolving 1 g in 100 ml distilled water and stirring at 30 °C for 60 min with a magnetic stirrer. The pH was adjusted to pH 7.0 with 1 M NaOH. The solution was poured into a 250 ml measuring cylinder, and volume V_o_ was taken. It was homogenized for 30 s, transferred immediately into a 250 ml measuring cylinder, and volume V_T_ was taken. The solution was allowed to stand for 30 min at room temperature, and volume V_t_ was taken.

$$\% \;{\text{Foam}}\;{\text{expansion}} = \left( {\left[ {{\text{V}}_{{\text{T}}} {-}{\text{V}}_{{\text{o}}} } \right]/{\text{V}}_{{\text{o}}} } \right) \times 100,$$

$$\% \;{\text{Foam}}\;{\text{stability}} = \left( {\left[ {{\text{V}}_{{\text{t}}} {-}{\text{V}}_{{\text{o}}} } \right]/{\text{V}}_{{\text{o}}} } \right) \times 100.$$

#### Emulsifying properties of GKPH

Emulsifying properties of GKPHs, including emulsifying activity index (EAI) and emulsion stability index (ESI), were determined as explained by Noman et al. (2019). A 0.5% and 1% (6 ml) of GKPHs with 10 ml of olein oil were mixed and homogenized for 2 min at 2000 rpm. From the bottom of the emulsion, 50 μl was taken and diluted with 5 ml of sodium dodecyl sulfate solution (0.1%). The absorbance of the solutions was measured at 500 nm after 0 and 10 min using a UV-spectrophotometer. Emulsifying properties were calculated thus:

$${\text{EAI}}\;\left( {{\text{m}}^{2} /{\text{g}}} \right) = \left( {2 \times 2.303 \times {\text{A}}_{0} } \right)/(\upphi \times {\text{S}}).$$

$${\text{ESI}}\;\left( {\min } \right) = \left( {{\text{A}}_{0} \times 10} \right)/({\text{A}}_{0} {-}{\text{A}}_{10} ).$$

where Ф = oil volume fraction (0.25); S = weight of sample.

### Statistical analysis

Results were demonstrated as mean ± standard deviation (SD). Statistical analyses were implemented using GraphPad software, Prism 5. Results were subjected to a one-way analysis of variance (ANOVA) followed by Tukey post hoc test to determine significant statistical differences between means. Differences were considered significant with *p* < 0.05.

## Results

### Proximate analysis

Chemical analysis of *G. kola* fresh seed revealed that it has relatively low crude protein content (4.97 ± 0.05%) compared to carbohydrate content (nitrogen-free extract) (43.15 ± 0.42%) and moisture content (45.96 ± 0.58%). Fat, ash and crude fibre in fresh *G. kola* seeds were 1.32 ± 0.06, 3.36 ± 0.01 and 1.25 ± 0.06%, respectively. However, GKPI only contained crude protein, ash and moisture alone with values of 76.35 ± 0.35, 6.43 ± 0.2 and 17.66 ± 0.39%, respectively.

### Total protein content

GKPI obtained by alkaline solubilization, and acid precipitation showed an increased protein content compared to the crude extract as shown in Table 1. The peptide contents of GKPHs is also shown in Table 1.

**Table 1:** Protein concentration of *G. kola* seed extract, protein isolate and enzymatic hydrolysates

Sample	Protein concentration (mg/ml)
Crude extract	32.70 ± 0.57
Protein isolate	120.86 ± 1.85
Protein hydrolysate	
1:8 (E:S, v/v)	2.47 ± 0.07
1:16 (E:S, v/v)	5.70 ± 0.09
1:32 (E:S, v/v)	11.34 ± 0.14

Results are expressed as mean ± SD of three trials (N = 3)

### Physicochemical characterization of GKPHs

#### Degree of enzymatic hydrolysis (DH) of GKPHs

**Fig. 1:**
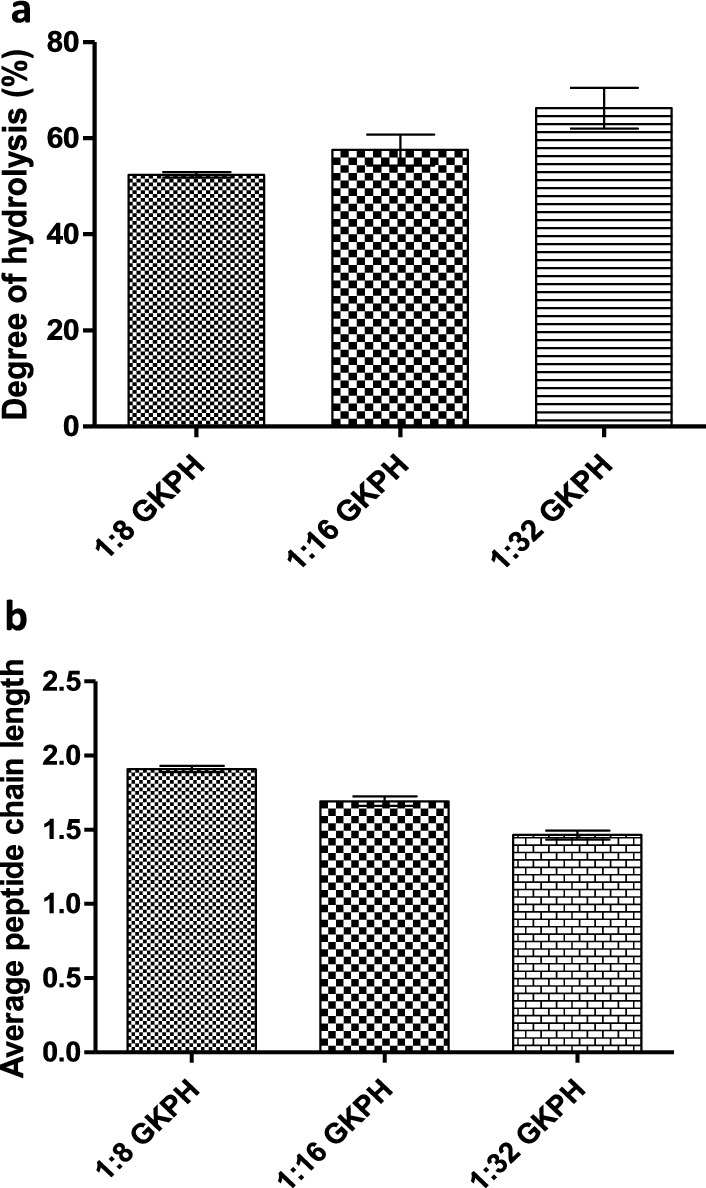
**a** Degree of enzymatic hydrolysis of *Garcinia kola* seed enzymatic hydrolysates (GKPHs), **b** Average peptide chain length of *Garcinia kola* seed enzymatic hydrolysates (GKPHs) at different enzyme: substrate ratio. Results are expressed as mean ± SD of three trials (N = 3)

GKPHs produced at 1:32 (E:S ratio) had the highest DH of 66.27% compared to others which were 52.38% and 57.55% for hydrolysates produced at 1:8 and 1:16 E:S ratio, respectively, as depicted in Fig. 1a. Result of average peptide chain length of GKPHs is depicted in Fig. 1b. Average peptide chain length was calculated from the degree of hydrolysis value, thus GKPH produced at 1:32 presented the lowest PCL (1.47), while GKPH produced at 1:8 (E:S ratio) showed the highest PCL of 1.91 (Fig. 1b).

### Amino acid composition of GKPH

The amino acid content of GKPI and GKPH (1:16 E:S) is shown in Table 2. Results revealed that the dominant essential amino acids were leucine—8.55 and 9.54 g/100 g, lysine—5.36 and 6.28 g/100 g and arginine—5.33 and 6.54 g/100 g, respectively in GKPI and GKPH. Glutamic acid and aspartic acid were the predominant non-essential amino acid in both GKPI and GKPH.

**Table 2:** Amino acid composition of *Garcinia kola* seed protein isolate and enzymatic hydrolysate

Amino acids	Concentration (g/100 g protein)	
	Protein isolate	Enzymatic hydrolysate
Leucine	8.55	9.54
Lysine	5.36	6.28
Isoleucine	3.63	4.06
Phenylalanine	4.35	3.99
Tryptophan	2.13	2.47
Valine	4.79	4.30
Methionine	2.06	2.38
Arginine	5.33	6.54
Histidine	1.85	2.62
Threonine	4.13	3.64
Proline	3.65	5.08
Serine	3.35	4.27
Tyrosine	3.96	3.61
Glycine	4.06	3.42
Cystine	1.82	1.82
Alanine	4.28	5.01
Glutamic acid	14.00	16.35
Aspartic acid	9.02	10.08
Essential amino acids	42.18	45.82
Non- essential amino acids	44.12	49.64
Hydrophobic amino acids	37.50	40.25
Hydrophilic amino acids	48.82	55.21
Basic amino acids	12.54	15.44
Acidic amino acids	23.02	26.43
Aromatic amino acids	10.41	10.07

### In vitro antioxidant activities of GKPHs

#### DPPH free radical scavenging of GKPH

**Fig. 2:**
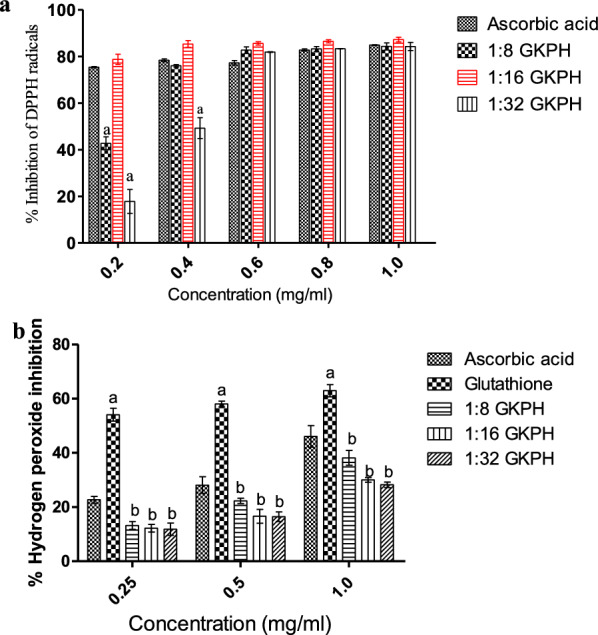
Antioxidant activities of enzymatic hydrolysates of *Garcinia kola* seeds (**a** DPPH radical scavenging activity, **b** hydrogen peroxide scavenging activity. Results are expressed as mean ± SD of three trials (N = 3). ^a^p < 0.05 as compared to ascorbic acid and ^b^p < 0.05 as compared to glutathione

The DPPH radical scavenging activity of GKPHs is represented in Fig. 2a. GKPHs inhibited the formation of DPPH free radicals in a concentration dependent manner, with GKPH produced at 1:16 displaying a maximum DPPH scavenging ability of 87.24 ± 0.10% at 1 mg/ml concentration, which was significantly similar to ascorbic acid at 1 mg/ml (84.91 ± 0.13%).

#### Iron (II) chelating activity of GKPH

GKPHs produced at 1:8 and 1:32 (E:S) ratio showed no chelating ability, whereas only those produced at 1:16 (E:S) ratio showed a chelating ability of 17.06 ± 0.05 mg/ml, as shown in Table 3.

**Table 3:** Chelation of iron (II) by *Garcinia kola* seed enzymatic hydrolysates (GKPHs)

Sample	% Inhibition		
	1 mg/ml	0.5 mg/ml	0.25 mg/ml
AA	21.79 ± 0.04	12.36 ± 0.03	8.19 ± 0.07
GSH	16.70 ± 0.01^a^	11.80 ± 0.02	7.37 ± 0.01
1:16 GKPH	17.06 ± 0.05^a^	2.93 ± 0.03^ab^	0

Results are expressed as mean ± SD of three trials, N = 3

^a^p < 0.05 as compared to AA (ascorbic acid)

^b^p < 0.05 as compared to GSH (glutathione)

#### Hydrogen peroxide scavenging activity of GKPH

GKPHs scavenged H_2_O_2_ in a concentration-dependent manner, as shown in Fig. 2b. GKPH produced at 1:8 (E:S) ratio had the highest H_2_O_2_-scavenging activity of 38.17% at 1 mg/ml.

### In vitro anti-inflammatory activities of *G. kola* seed enzymatic hydrolysates (GKPHs)

#### Human red blood cell (HRBC) membrane stabilization activity of GKPH

GKPHs inhibited HBRC lysis induced by water. This is shown by the percentage hemolysis inhibition presented in Table 4. GKPH obtained at 1:16 (E:S) ratio showed the highest percentage hemolysis inhibition, 84.49% at maximum concentration, 1 mg/ml, which compared well with the standard acetylsalicylic acid (77.82%).

**Table 4:** Percentage hemolysis inhibition by *Garcinia kola* seed enzymatic hydrolysates (GKPHs) in HRBCs membrane stabilization test

Sample	0.25 mg/ml	% Inhibition	0.50 mg/ml	% Inhibition	1.0 mg/ml	% Inhibition
Control	0.78 ± 0.02	–	0.78 ± 0.02	–	0.78 ± 0.02	–
ASA	0.54 ± 0.02^a^	31.28	0.33 ± 0.01^a^	57.63	0.17 ± 0.02^a^	77.82
1:8 GKPH	0.45 ± 0.08^ab^	43.01	0.39 ± 0.01^a^	49.87	0.14 ± 0.07^a^	81.54
1:16 GKPH	0.30 ± 0.03^ab^	62.18	0.14 ± 0.05^ab^	82.37	0.12 ± 0.07^a^	84.49
1:32 GKPH	0.63 ± 0.08^ab^	19.55	0.31 ± 0.03^a^	60.77	0.20 ± 0.01^a^	74.36

All values are expressed as mean ± SD, N = 3

^a^p < 0.05 as compared to contro

^b^p < 0.05 as compared to ASA (acetylsalicylic acid)

#### Protein denaturation inhibition activity of GKPH

The result of percentage inhibition of protein denaturation of GKPHs is shown in Table 5. GKPHs (1 mg/ml) showed maximum percentage denaturation inhibition of 54.39%, 53.36% and 51.48% for hydrolysates produced at 1:8, 1:16 and 1:32 (E:S) ratio, respectively, whereas, the maximum percentage denaturation inhibition of acetylsalicylic acid (ASA) was found to be 54.62%.

**Table 5:** Percentage protein denaturation inhibition by *Garcinia kola* seed enzymatic hydrolysate (GKPH) in egg albumin protein denaturation test

Sample	0.25 mg/ml	% Inhibition	0.50 mg/ml	% Inhibition	1.0 mg/ml	% Inhibition
Control	1.27 ± 0.02	–	1.27 ± 0.02	–	1.27 ± 0.02	–
ASA	0.80 ± 0.03^a^	37.07	0.67 ± 0.09^a^	46.75	0.58 ± 0.01^a^	54.62
1:8 GKPH	0.79 ± 0.03^a^	37.9	0.68 ± 0.02^a^	47.46	0.58 ± 0.04^a^	54.39
1:16 GKPH	1.05 ± 0.08^ab^	17.67	0.71 ± 0.07^a^	44.2	0.59 ± 0.01^a^	53.36
1:32 GKPH	1.10 ± 0.08^ab^	13.81	0.76 ± 0.05^ab^	40.46	0.62 ± 0.03^ab^	51.48

All values are expressed as mean ± SD, N = 3

^a^p < 0.05 as compared to control

^b^p < 0.05 as compared to ASA (acetylsalicylic acid)

### Functional properties of *G. kola* seed enzymatic hydrolysate (GKPH)

GKPHs with the overall highest in vitro antioxidant activities were found to be the hydrolysate produced at 1:16 (E:S) ratio; hence, it was investigated for functional properties.

#### Solubility of *G. kola* seed enzymatic hydrolysates

**Fig. 3:**
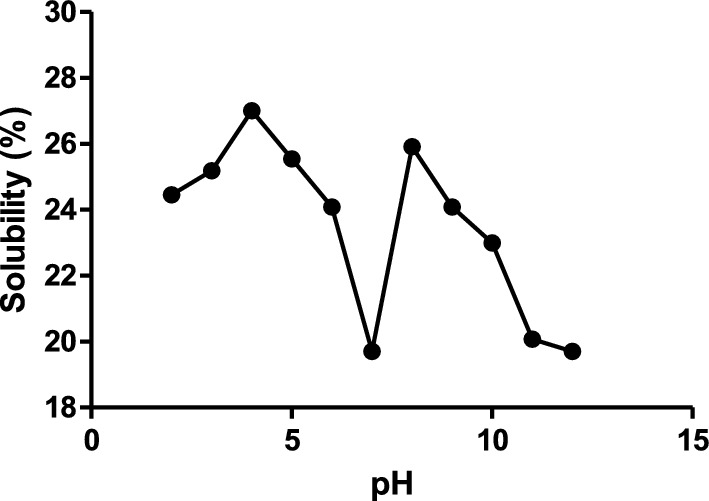
Solubility of *Garcinia kola* seed enzymatic hydrolysates obtained at 1:16 E:S ratio

The solubility profile of GKPH is presented in Fig. 3. GKPH was found to be soluble across the pH range (pH 2–12), as shown in Fig. 3. The hydrolysate showed maximum solubility at pH 4 (27.00%) and minimum at pH 7 (19.77%).

#### Foaming effects of *G. kola* seed enzymatic hydrolysates

GKPH showed a high foaming capacity of 50%, while the foaming stability after 30 min was 9.38% compared to that of the protein isolate, which was 5.88% for both foam expansion and stability (Table 6).

**Table 6:** Foaming properties of *Garcinia kola* seed protein isolate and enzymatic hydrolysate

Sample	Foam expansion (%)	Foam stability (%)
GKPI	5.88	5.88
GKPH	50.00	9.38

#### Emulsifying properties of *G. kola* seed enzymatic hydrolysates

The ability of GKPH to form an emulsion with triolein oil determined using emulsifying ability index (EAI) and emulsifying stability index (ESI) was recorded in Table 7. The EAI and ESI of GKPH were concentration dependent as they increased from 3.87 m^2^/g and 0.58 min (0.5 mg/ml) to 24.14 m^2^/g and 13.10 min (1.0 mg/ml), respectively.

**Table 7:** Emulsifying properties of *Garcinia kola* seed enzymatic hydrolysate

Concentration (mg/ml)	EAI (m^2^/g)	ESI (min)
0.5	3.87	0.58
1.0	24.14	13.10

*EAI* emulsifying activity index, *ESI* emulsifying stability index

## Discussion

Protein hydrolysates obtained from natural products under controlled conditions are safe alternatives to synthetic products which have been linked to several side effects (Sang et al. 2019). Naturally occurring peptides and those derived from protein hydrolysis are considered as novel and potential dietary ingredients to promote human health (Osukoya et al. 2020) as they have been recorded to possess certain potent bioactivities including antioxidant, antimicrobial, immunomodulatory, anti-inflammatory, anti-hypertensive, anti-human immunodeficiency virus (anti-HIV) and opioid activities (Chauhan and Kanwar 2020).

Chemical analysis of *G. kola* fresh seed revealed that the seed has relatively low crude protein content compared to carbohydrate content (nitrogen-free extract) and moisture content. A similar result from *G. kola* fresh seeds (Asaolu 2003; Odebunmi et al. 2009) and the dried fruit (Ea 2013; Esiegwu and Udedibie 2009). On the other hand, the GKPI showed only presence of crude protein, ash and moisture alone, confirming it as a protein isolate.

The higher protein content exhibited by GKPI when compared with *G. kola* crude extract indicates that the protein isolate is the refined form of protein products with a reduction in non-protein substances, hence containing the greatest concentration of protein. However, GKPHs produced at 1:8, 1:16 and 1:32 showed lower protein concentration indicating that peptide bond has been cleaved, hence reducing the amount of peptide nitrogen that interacted with copper [II] ions in the Lowry method.

DH is the proportion of occurrence of protein degradation. It is the amount of cleaved peptide bonds in a protein hydrolysate. The extent of a protein’s enzymatic hydrolysis depends on the protease enzyme’s capability to hydrolyze peptide bonds. Other factors affecting DH are enzyme: substrate ratio, temperature, pH, duration of hydrolysis, and solid: liquid ratio (Islam et al. 2021). In this study, the only factor that varied was enzyme: substrate ratio, and DH increased as the substrate concentration increased (at constant enzyme concentration). This is similar to the report by Shu et al. (2016), in which DH of goat milk hydrolysis by casein increased with increasing substrate concentration to a certain level. Generally, reaction rate is increased at increasing substrate concentration to a certain level at optimal enzyme concentration, temperature and pH because more substrate molecules are able to interact with enzyme (Juárez-Enríquez et al. 2022). Dada et al. (2023) also reported that the DH of trypsin hydrolysates of *Artocarpus altilis* protein increased as substrate concentration is increased. The increase in DH with increasing substrate concentration may be due to the production of inhibitory peptides during hydrolysis at low substrate concentration (and high concentration of enzyme), leading to a decrease in enzyme activity, shown by a low DH. Increasing the concentration of substrate caused a reduction in the inhibitory effect of the inhibitory peptides, leading to an increase in DH at high substrate concentrations (Dada et al. 2023; Deng et al. 2018). PCL is an essential index in determining the properties of protein hydrolysates, and it is inversely proportional to DH. Thus, GKPH with the highest DH had the lowest PCL value and vice versa. PCL can also be influenced by extraction conditions and exposure of terminal amino groups. Aside from functional properties, another factor that govern the absorption rate of protein hydrolysates (by that, its nutritional value) is PCL (Pankyamma et al. 2019).

Amino acid analysis is used to ascertain the amino acid contents of any protein-containing sample. In this study, the result of amino acid composition of GKPI and GKPH revealed that glutamate and aspartate were the most prominent. This is probably by virtue of conversion of glutamine and asparagine in the quantification process due to the instability of the amide side chains of glutamine and asparagine to acid hydrolysis (6 M HCl, 105 °C, 22 h) used to determine protein-bound amino acids (Johns and Hertzler 2021). However, the dominant essential amino acids were leucine, lysine and arginine in both GKPI and GKPH. The total amino acid of the GKPI was 86.32 g/100 g protein, while that of GKPH was 95.46 g/100 g protein. The percentage of total essential amino acids was 48.46% and 47.99%, respectively, for GKPI and GKPH. Similar results were reported by Eleyinmi et al. (2006), who reported that the fraction of essential amino acids in *G. kola* seed was 35.81%, with lysine, leucine and valine being the most predominant. Also, Adeyeye et al. (2007) reported that the total amino acids in *G. kola* seed were found to be 112.90 mg/g protein, with essential amino acids amounting to 47.05%.

Antioxidant activities of hydrolyzed proteins depend on the protease used and the conditions of hydrolysis (Osukoya et al. 2021). DPPH free radical is stable (in powder form) with purple color, but turns yellowish/colorless when scavenged. The DPPH assay uses this characteristics to show free radical scavenging activity which is measured spectrophotometrically. DPPH is reduced to DPPH-H in the presence of antioxidants, which consequently causes a decrease in absorbance, indicated by the extent of discoloration. In the present study, GKPHs inhibited the formation of DPPH free radicals in a concentration-dependent manner. These findings compare well with the study by Osukoya et al. (2021) that reported that *Monodora myristica* protein hydrolysates exhibited radical scavenging potential in a concentration-dependent manner based on the protease used and duration of hydrolysis. Several previous studies showed that food-derived protein hydrolysates were capable of scavenging DPPH free radicals such as hydrolysates from Azufrado bean (Bahi et al. 2016), African breadfruit peptide hydrolysates (Osukoya et al. 2020), sole fish skin and scale gelatin hydrolysates (Pankyamma et al. 2019), and protein hydrolysates from grass turtle (Islam et al. 2021).

Chelating agents are organic or inorganic compounds that can bind toxic metal ions [e.g. iron (II)]. The binding of chelating agents to these toxic metals results in complex structures that are readily expelled, thus eliminating them from intra- or extracellular spaces (Flora and Pachauri 2010). The ability of any molecule to chelate iron (II) contributes significantly to antioxidant activities because of the involvement of iron (II) in lipid peroxidation and also in hydroxyl radical (OH^−^) production (Sarbon et al. 2018). Of all GKPHs produced at different E:S ratios, only those produced at 1:16 (E:S) ratio showed a relative chelating ability. This could be a result of changes in the size, quantity, and composition of free amino acids and small peptides (Osukoya et al. 2021). Iron chelating activity has been established in various protein hydrolysates, including African breadfruit peptide hydrolysates (Osukoya et al. 2021) and sole fish skin and scale gelatin hydrolysates (Pankyamma et al. 2019).

H_2_O_2_ is an important ROS owing to it being able to infiltrate biological membranes (Chen et al. 2017). This causes severe damage to biomolecules and initiates oxidative stress. The measure of the disappearance of H_2_O_2_ at 230 nm caused by an antioxidant molecule is employed in ascertaining the antioxidant properties of the molecule. In this study, GKPHs caused a decrease in the appearance of H_2_O_2_. There are reports on H_2_O_2_ scavenging activities of several hydrolysates, such as hydrolysates of *Arca Subcrenata* prepared with three proteases (neutrase, alcalase and papain) (Song et al. 2008), hydrolysates of *Mormyrus rume* muscle protein (Jerome and Olayemi 2019) and rice protein hydrolysates (Zhang et al. 2020).

Inflammation is a protective biological response of vascular tissues to toxins or harmful stimuli and the initiation of healing processes (Javed et al. 2020). HRBC membrane stabilization prevents lytic enzymes and inflammatory mediator discharges; hence, HRBC membrane stabilization tests can be used to evaluate anti-inflammatory activities. In this study, hemolysis, which is the lysis or rupturing of RBCs, was induced by hyposaline (hypotonic solution). This is connected with the buildup of fluid within the cell resulting in cell rupturing. GKPHs inhibited hemolysis in HRBC membrane stabilization test. Similar results have been reported in some protein hydrolysates, such as trypsin protein hydrolysate of *Harpa ventricosa* (visceral mass of gastropod) (Joshi et al. 2016), *Charibdis natator* papain hydrolysates (Balde et al. 2021) and whey trypsin hydrolysate (Embiriekah et al. 2018).

Protein denaturation, membrane alteration and vascular permeability are the major factors that result in inflammation. Protein denaturation is a process whereby the application of external force or compounds such as strong acid/base, inorganic salt or heat causes the recession of their secondary and tertiary structure (Leelaprakash and Dass 2011), and thus their biological functions (protein structure dictates function). Hence, as part of the investigation of the anti-inflammatory properties of GKPHs, the ability of the hydrolysates to inhibit heat-induced protein denaturation was studied. GKPHs showed significant percentage protein denaturation inhibition of 54.39%, 53.36% and 51.48% for hydrolysates produced at 1:8, 1:16 and 1:32 (E:S) ratio, respectively, comparably with the standard ASA. This result is similar to the reports of Balde et al. (2021) who reported that *Charibdis natator* papain protein hydrolysates had highest albumin denaturation inhibition of 78.55 ± 1.36%, whereas 50% inhibition at 5.38 ± 0.02 mg/ml was reported for trypsin protein hydrolysate of *Harpa ventricosa* (Joshi et al. 2016).

The functional properties of proteins depend on their capacities to go into solution, and this is enhanced by enzymatic hydrolysis, which influences the molecular size and interactions with water (Osukoya et al. 2021). Protein solubility is the main characteristic in selecting proteins for use in foods and beverages. Solubility is the most critical functional property of proteins since it significantly influences other functional properties, such as foaming capacity and emulsifying properties (Pankyamma et al. 2019). This is attributed to protein structure laying the groundwork for its relationship with other molecules and, ultimately, determining its function. Protein solubility is generally affected by pH; hence, in this study, the solubility of GKPH was evaluated across pH 2–12. GKPH produced at 1:16 was found to be soluble across all pH range. Protein solubility has been established for several protein hydrolysates, such as protein hydrolysates of *Monodora myristica* produced with pepsin, pancreatin and trypsin (Osukoya et al. 2021) and protein hydrolysates of *Chinemys reevesii* (Islam et al. 2021).

Food foam is formed when air bubbles are dispersed in water. Protein stabilizes foam by forming a protective wall around the air bubbles in the foam (so that it does not collapse) with its hydrophilicc amino acid (that binds to water) and its hydrophobic amino acid (that holds the air). Three factors that determine the foaming capacity of a protein are transportation, penetration, and reorganization of molecules at the air–water interface (Pankyamma et al. 2019). In general, protein foaming capacity is related to molecular weight since hydrolysates with higher molecular weights foam better, while micro-peptides rarely have the vigour to sustain a stable foam (Osukoya et al. 2021). GKPH exhibited a high foaming capacity and foaming stability. This can be linked to the fact that the hydrolysate has more residues which would have more reactive ends (hydrophilic groups) than the protein isolates due to its compositions of peptide fragments with more C- and N-terminus in addition to hydrophilic side chains.

Emulsification is the process of breaking down fat into smaller components, making it easy for enzymes to function and digest food. GKPH presented a concentration-dependent emulsifying properties. Emulsifying properties may be affected by the surface hydrophobicity and solubility of the protein hydrolysate. Some studies have shown that a highly soluble protein would have an excellent emulsifying ability (Horax et al. 2011). Nalinanon et al. (2011) found the same effect for protein hydrolysate from ornate threadfin bream at different levels of degree of hydrolysis using pepsin. Excessive hydrolysis has been reported to decrease the emulsification characteristics of protein hydrolysates (Kristinsson and Rasco 2000). Therefore, it can be stated that the molecular properties, particularly peptide size and protein concentration, could affect the emulsifying properties of protein hydrolysates.

In summary, this study showed that *Garcinia kola* seed enzymatic hydrolysate obtained with pepsin scavenged free radicals (including DPPH and H_2_O_2_) and acted as a chelating agent; therefore, the enzymatic hydrolysates possess excellent in vitro antioxidant activities. In addition, *G. kola* seed pepsin enzymatic hydrolysate stabilized the red blood cell membrane and inhibited egg protein denaturation, revealing its anti-inflammatory potential. Increasing substrate concentration at constant enzyme concentration caused an increase in the degree of hydrolysis but did not necessarily increase the bioactivity of *G. kola* enzymatic (pepsin) hydrolysates. The pepsin enzymatic hydrolysate produced at 1:16 enzyme: substrate ratio exhibited the overall highest antioxidant and anti-inflammatory activities. This enzymatic hydrolysate possessed excellent solubility, foaming and emulsifying properties. Hence, *G. kola* pepsin enzymatic hydrolysates have the potential to be developed as food ingredients and as therapeutics in pharmaceutical industries to manage oxidative stress and inflammation disorders.

## Data Availability

The datasets generated during and/or analysed during the current study are available from the corresponding author on reasonable request.
